# First report of granulomatous mastitis associated with Sjögren’s syndrome

**DOI:** 10.1186/1477-7819-11-268

**Published:** 2013-10-10

**Authors:** Christel Letourneux, Pierre Diemunsch, Anne-Sophie Korganow, Cherif Youssef Akladios, Jean-Pierre Bellocq, Carole Mathelin

**Affiliations:** 1 Breast Diseases Center, Hautepierre Hospital, University Hospital (CHRU), avenue Molière, Strasbourg cedex, 67200, France; 2 Department of Anaesthesiogy, Hautepierre Hospital, University Hospital (CHRU), avenue Molière, Strasbourg cedex, 67200, France; 3 Department of Clinical Immunology and Internal Medicine, Civil Hospital, 1, place de l’Hôpital, BP 426, Strasbourg cedex, 67091, France; 4 Department of Pathology, Hautepierre Hospital, University Hospital (CHRU), avenue Molière, Strasbourg cedex, 67200, France

**Keywords:** Granulomatous mastitis, Sjögren’s syndrome, Benign breast disease

## Abstract

Granulomatous mastitis is a rare and often considered as idiopathic disease. However, clinical examination and thorough diagnostic investigations have to be carried out in order to identify cases that are secondary to infections or systemic diseases since these forms may be cured with appropriate etiologic treatment. To the best of our knowledge, this report is the first to describe the association of granulomatous mastitis with Sjögren’s syndrome. We discuss the clinical, pathological and therapeutic implications of this association.

## Background

Granulomatous mastitis (GM) is a chronic inflammatory disease of the mammary gland, characterized by recurrent inflammatory breast tumors of varying sizes, usually unilateral, and without nipple retraction [1]. These tumors can fistulize with subsequent infection. GMs were studied by Kessler and Wolloch in 1972 [2]. Only a few hundred cases have been reported worldwide [3,4], reflecting the low incidence of this disease.

The images obtained with mammography, ultrasound and magnetic resonance imaging (MRI) are nonspecific and correspond to heterogeneous masses of variable sizes with irregular shapes, sometimes confluent, and without atypical microcalcifications allowing for diagnosis confirmation [5,6]. Histological examination (microbiopsy or surgical specimen) is required to obtain a definitive diagnosis. Within these nodules there is generally a breast inflammatory infiltrate rich in lymphocytes (B and T), plasma cells, neutrophils and histiocytes. Interestingly, there is sometimes a diffuse infiltration of immunoglobulin (Ig) G4 + cell type, associated with high serum IgG4 concentration [5]. Sometimes an invasion of the lobules by giant cells associated with microabscesses can be observed, and outbreaks of noncaseating necrosis in acini and intralobular ducts can be associated with the cellular inflammatory infiltrate [7].

Most of the time, the GM is considered idiopathic but there are cases of GMs secondary to infection or systemic diseases. A wide range of microorganisms have been implicated, and fungi, parasites, as well as bacteria have all been reported to be causal in GM. However, involved microorganisms are mainly corynebacteria and specifically *Corynebacterium kroppenstedtii*[8], and *Propionibacterium acnes*[8], *Mycobacterium tuberculosis*[9] and *Brucella melitensis*[10]. Thus, a higher prevalence of secondary GMs has been observed in Mediterranean and Asian countries (China and Malaysia) [11], probably because of endemic tuberculosis or brucellosis in these regions.

Secondary GMs have also been reported in association with sarcoidosis [12], Wegener’s disease [13], systemic lupus erythematosus [11], erythema nodosum [14] and isolated oligoarthritis [15]. Such GMs that are secondary to connectivites have usually a favorable outcome when on corticosteroids [1]. Secondary GMs occur at different ages depending on the underlying disease with extremes ranging from 11 to 83 years. Idiopathic GMs are more common in women of childbearing age, taking oral contraceptives or close to a period of childbirth or breastfeeding, which raises the question of hormonal influence, though it has never been proven [1].

We would like to report a case of association of GM with Sjögren’s syndrome (SjS). We will review the clinical, diagnostic and scalable particularities of this association that has, to the best of our knowledge, not been described before.

## Case presentation

### Observation

Mrs NW, age 68, menopausal for 20 years, was sent in May 2008 to the Breast Diseases Center of Strasbourg University Hospital for the management of a ‘recurrent abscess of the left breast’ that had been developing since July 2007. She had suffered four inflammatory lesions spreading successively in the internal and then external para-areolar area of her left breast. Neither hyperthermia nor biologic infectious syndrome was associated with these episodes.

Her past medical history was positive for xerostomia still treated with a saliva substitute. Our patient neither smoked nor drank alcohol. Regarding her family, one of her sisters was treated for nodosa periarthritis associated with mononeuritis and SjS, whereas her other sister had died at age 64 after a pulmonary embolism. Also, a cousin had had breast cancer. Twenty years ago, between 1989 and 1991, Mrs NW presented with dysesthesia of the upper and lower limbs with osteotendinous reflexes and sensorimotor deficit.

The electromyogram (EMG) and peripheral nerve biopsy were indicative of a mononeuritis. The brain MRI uncovered two lesions of demyelination. Biological investigation of autoimmunity proved positive for antinuclear antibodies (ANA) of speckled pattern and anti-SS-A and SS-B antibodies. The findings included also a rheumatoid factor and polyclonal hyperglobulinemia. By contrast, antineutrophil cytoplasmic antibodies (ANCAs) were negative and IgG4s were not detected in the blood.

Treatment with corticosteroids and plasma exchange did not improve her sensory disorders.

**Figure 1:**
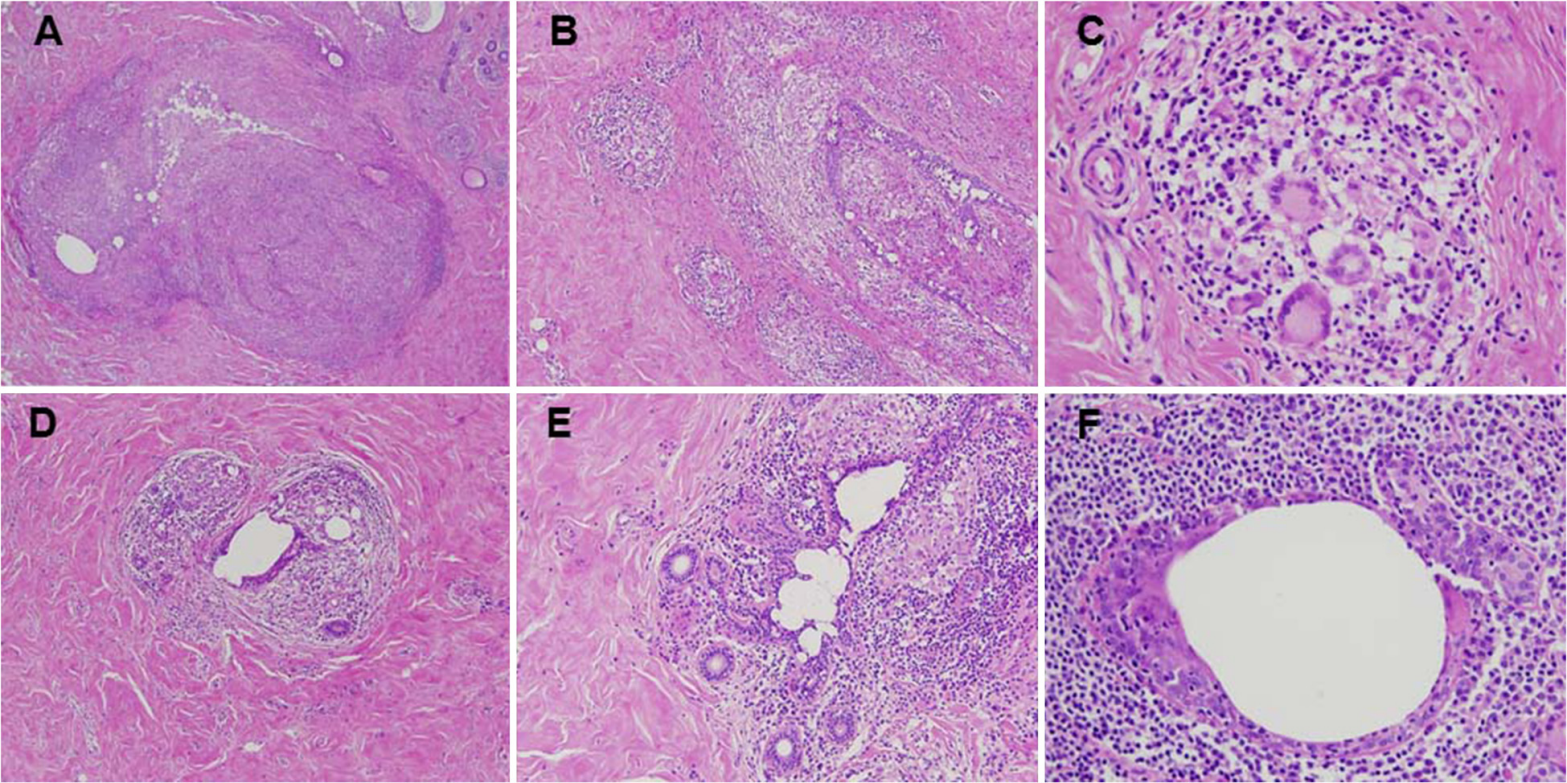
**Histological sections of breast nodules examined after hematoxylin and eosin staining. (A)** Chronic granulomatous inflammation of nodular arrangement (×40). **(B and C)** Multiple confluent granulomas rich in multinucleated giant cells (B ×100 and C ×400). **(D and E)** Lobulite lymphocytic and granulomatous (D ×100 and E ×200). **(F)** Periductal inflammation rich in neutrophils, macrophages and lymphocytes (×400).

All of her four breast lesions were successively treated by surgical excisions (in July, September and December 2007 and March 2008). Histological examination of the surgical specimens concluded that the lesions of chronic galactophoritis were associated with granulomatous inflammatory lesions (Figure 1). The search for *Corynebacterium* and *Mycobacterium tuberculosis* proved negative on specific media. Superinfection of the lesions by multisensitive Staphylococcus led to order long-term treatment with pristinamycin, amoxicillin and clavulanate and later ciprofloxacin, but without clinical improvement.

**Figure 2:**
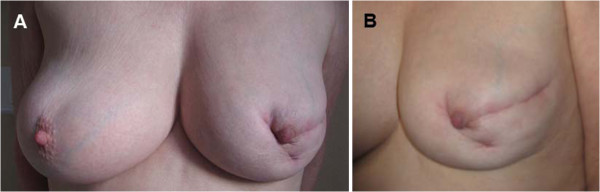
**Photography of the breasts. (A)** Breast face. **(B)** Left breast in profile showing a radial and periareolar scar in the mid-outer quadrant and a periareolar scar and an internal fistula in the inferolateral quadrant. No lesions were noted on the right breast.

When she first consulted in our Breast Diseases Center, the clinical examination of Mrs NW was remarkable for a radial and retractable scar in the mid-outer quadrant of her left breast associated with a fistula in the inferolateral quadrant (Figure 2). No lesion of the right breast was observed. Mammography and breast ultrasound showed no lesion suggestive of breast cancer but typical aspects of the scarred areas. Carcinoma antigen (CA) 15.3 assay was normal at 13.8 IU/ml. The rest of the examination revealed xerophtalmia without xerostomia (a positive Schirmer test), Raynaud’s syndrome in the upper limbs, discrete bilateral basal crackling lung rales and a sensory polyneuropathy in the upper and lower limbs.

**Figure 3:**
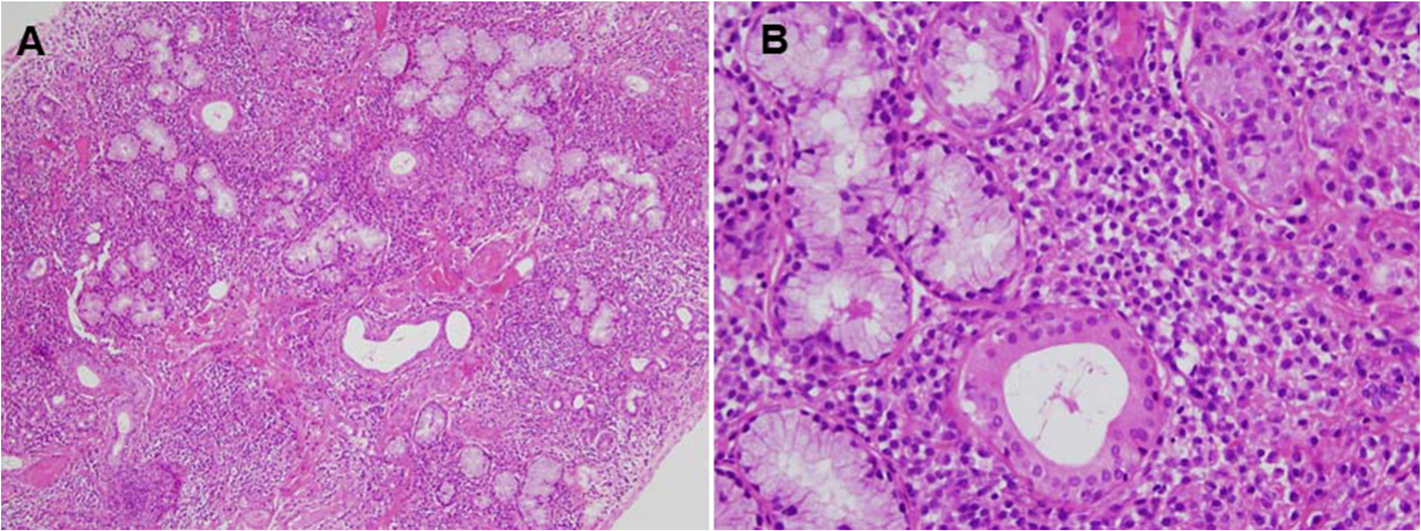
**Histological biopsies of salivary glands examined after hematoxylin and eosin staining. (A)** Note the presence of multiple inflammatory foci (focus: nodular clusters of at least 50 cells) associated with collagen fibrosis, corresponding to a level 4 according to the classification of Chisholm and Mason (×100). **(B)** It shows an intense infiltrate of chronic inflammatory lymphocytes and plasma cells, interstitial, especially surrounding the acini and the ducts (×400).

Salivary gland biopsy had found a lymphoplasmacytic infiltrate of grade IV according to Chisholm’s classification [16] (Figure 3). The EMG confirmed a mononeuritis, the brain MRI highlighted lesions suggestive of demyelination with inflammatory aspects, and chest scan showed diffuse bronchiectasis-type lung damage. Autoimmunity evaluation results were superimposable to those obtained twenty years earlier. The diagnosis of SjS with its neurological complications was established on clinical (ocular symptoms, a positive Schirmer test), histological (biopsy of the salivary glands) and biological arguments (positiveness of ANA and of anti SS-A and SS-B antibodies). Reiterated inflammatory breast lesions simultaneously associated with histological granulomas had led to the diagnosis of GM. The diagnosis of a GM secondary to SjS was therefore hypothesized. Local treatment and systemic treatment with corticosteroids allowed for the healing of the breast lesions without further surgery. Four years later, clinical stability of the SjS was confirmed without any new outbreak of GM.

## Discussion

This article describes the case of a patient presenting simultaneously with both GM and SjS. SjS is an autoimmune disease characterized by chronic lymphocytic infiltration and destruction of exocrine glands [17]. Patients complain of xerophthalmia, xerostomia, xerodermia, xerorhinia and vaginal dryness. Nonglandular manifestations are reported in one case in two and appear to be related to lymphocytic infiltration and/or leukocytoclastic vasculitis. Our patient had multiple extraglandular manifestations involving the neurological system together with a sensory polyneuropathy, and respiratory tract with a bronchiectasis. The prevalence of SjS in the general population is 0.1 to 0.6% with a female preponderance and two frequency peaks according to age: one between 20 and 30 years and the other, more important, around 50 to 55 years. Our patient was 68 years old when she was referred to our Breast Diseases Center, but her disorders suggestive of SjS dated back to 20 years earlier. As in 70% of the cases of SjS, her biology revealed positive for anti-SS-B and anti-SS-A antibodies. A rheumatoid factor is present in 60% of the cases of SjS. Pure forms of SjSs have to be sorted from those associated with other systemic illness. Raynaud’s syndrome - that concomitantly affected our patient - is reported in nearly 20% of the cases of SjS.

When GM is diagnosed, the therapeutic approach requires first a thorough search for an etiological factor. Upon this first step, if infectious agents are identified as potentially causal, specific therapy (antibiotic-, antifungal- or antiparasitic-adjusted) is carried out. In cases where a systemic disease is associated or where the GM is recognized as idiopathic the treatment consists of corticosteroids.

As far as surgery is concerned, a recent study by Maffini *et al*. [18] showed that 50% of the patients had complete resolution of their symptoms under corticosteroids whereas the lesions in the other half remained stable. The authors therefore advocate surgical abstention. Also, surgery should not be the first-line approach because of its esthetic and psychological impact. Our patient was really concerned about the aspect of her left breast, deeply deformed by large and retractile scars. Furthermore, in the case of secondary MG, surgery is regularly followed by recurrences, should the underlying causal disease remain ignored and untreated.

A causal link between GM and a systemic disease is considered established when both pathologies developed simultaneously and recede once the specific treatment of the given disease had been initiated. Such a causal link has been reported for secondary GMs in association with sarcoidosis, Wegener’s disease, giant cell arteritis, periarteritis nodosa and certain isolated oligoarthritis. In the case of SjS, this causal link with GM is more difficult to establish firmly since SjS does not evolve in spurts and since no specific treatment for SjS has proven its complete efficiency so far [17]. In addition, from a histological point of view, there is no such thing as granulomatous-type reaction in the exocrine glands in the evolution of SjS. The exocrine glands are actually destroyed by the lymphocytic infiltrate and immune complexes. There are granulomas in GMs such as those found in sarcoidosis, rheumatoid arthritis or even the Wegener’s granulomatosis.

SjS, however, has been associated with granulomatous diseases such as granulomatous panniculitis [19], which corresponds to a skin invasion by noninfectious granulomas, curable with colchicine. Similarly, granuloma annulare may be associated with SjS [20]. Finally, the first description of three cases of SjS associated with granulomatous hepatitis was published in 2006 [21]. An infiltration by lymphoplasmocytes with histiocytes and epithelioid granulomas was found in the periportal and lobular part of the liver. These hepatic lesions were reduced under azathioprine and corticosteroid treatment.

In our observation, a combination of factors was strongly suggestive of a causal link between SjS and GM. Conversely, the patient’s age seems too old for idiopathic GM, which occurs more readily in the fourth decade and is frequently correlated with hormonal changes (pregnancy, lactation, oral contraceptives and so on). No hormonal taking was reported in this patient. Moreover, as far as her relatives were concerned, one of her sisters was followed for SjS associated with nodosa periarthritis, a disease that causes secondary GM. On the biological side, our patient had hyperglobulinemia at diagnosis of SjS, which was also observed in the GM. Finally, the existence of other granulomatous diseases (skin or liver) during the evolution of SjS indicates the possibility of developing inflammatory granulomas outside the exocrine glands.

## Conclusion

Our observation summarizes the medical history of a 68-year-old patient presenting with concomitant GM and SjS. To the best of our knowledge, this is the first report of this association. The surgical approach of the GM that was proposed as a first-line treatment proved inefficient and led to recurrences and severe esthetic sequels. The medical approach based on corticosteroids eventually solved both pathologies without further surgery.

This case stresses the fact that, in the presence of GM, the idiopathic nature of the disease should only be admitted after a careful search for a causal morbid association, particularly of infectious or systemic nature. Etiological treatment of the underlying pathology is associated with simultaneous healing of the breast illness. Surgery should be reserved as a second-line option only.

## Consent

Written informed consent was obtained from the patient for publication of this case report and any accompanying images. A copy of the written consent is available for review by the Editor-in-Chief of this journal.

## Abbreviations

ANA: antinuclear antibodies; GM: granulomatous mastitis; EMG: electromyogram; Ig: immunoglobulin; MRI: magnetic resonance imaging; SjS: Sjögren’s syndrome.

## Competing interests

The authors declared that they have no competing interests.

## Authors’ contributions

CL, CM, CYA and PD treated the breast disease and drafted the manuscript. JPB performed the pathological analysis and helped to draft the manuscript. ASK treated the Sjögren’s syndrome and helped to draft the manuscript. All authors read and approved the final manuscript.
